# The RNA world hypothesis: the worst theory of the early evolution of life (except for all the others)^a^

**DOI:** 10.1186/1745-6150-7-23

**Published:** 2012-07-13

**Authors:** Harold S Bernhardt

**Affiliations:** 1 Department of Biochemistry, University of Otago, P.O. Box 56, Dunedin, New Zealand

**Keywords:** RNA world hypothesis, Proteins first, Acidic pH, tRNA introns, Small ribozymes

## Abstract

The problems associated with the RNA world hypothesis are well known. In the following I discuss some of these difficulties, some of the alternative hypotheses that have been proposed, and some of the problems with these alternative models. From a biosynthetic – as well as, arguably, evolutionary – perspective, DNA is a modified RNA, and so the chicken-and-egg dilemma of “which came first?” boils down to a choice between RNA and protein. This is not just a question of cause and effect, but also one of statistical likelihood, as the chance of two such different types of macromolecule arising simultaneously would appear unlikely. The RNA world hypothesis is an example of a ‘top down’ (or should it be ‘present back’?) approach to early evolution: how can we simplify modern biological systems to give a plausible evolutionary pathway that preserves continuity of function? The discovery that RNA possesses catalytic ability provides a potential solution: a single macromolecule could have originally carried out both replication and catalysis. RNA – which constitutes the genome of RNA viruses, and catalyzes peptide synthesis on the ribosome – could have been both the chicken *and* the egg! However, the following objections have been raised to the RNA world hypothesis: (i) RNA is too complex a molecule to have arisen prebiotically; (ii) RNA is inherently unstable; (iii) catalysis is a relatively rare property of long RNA sequences only; and (iv) the catalytic repertoire of RNA is too limited. I will offer some possible responses to these objections in the light of work by our and other labs. Finally, I will critically discuss an alternative theory to the RNA world hypothesis known as ‘proteins first’, which holds that proteins either preceded RNA in evolution, or – at the very least – that proteins and RNA coevolved. I will argue that, while theoretically possible, such a hypothesis is probably unprovable, and that the RNA world hypothesis, although far from perfect or complete, is the best we currently have to help understand the backstory to contemporary biology.

**Reviewers:**

This article was reviewed by Eugene Koonin, Anthony Poole and Michael Yarus (nominated by Laura Landweber).

## Background

The problems associated with the RNA world hypothesis are well known, not least to its proponents [1,2]. In the following, I discuss some of these difficulties, some of the alternative hypotheses that have been proposed (including the ‘proteins first’ hypothesis), and some of the problems with these alternative models. As part of the discussion, I highlight the support provided to the RNA world concept by the discovery of some extremely small ribozymes. The activities of these provide support for proposals we have made previously for the identity of the first tRNA [3], for the origin of coded ribosomal protein synthesis [4], and for the evolution of an RNA world at acidic pH [5] (see also [6]). I also revisit the proposal for a replicase origin of the ribosome, and what has become the most commonly held model for the origin of tRNA.

In modern biological systems, the components of DNA are synthesized from RNA components [7], and it therefore makes sense to view DNA as a modified RNA. Similarly, the ribosome – the universal cellular machine that makes proteins – is composed mainly of RNA, and RNA is its active component, although there are indications that proteins may be playing an increasing role in some instances *e.g.*[8,9] (even in the case of nonribosomal peptide synthesis [10,11], the protein enzyme complexes that synthesize other proteins are of course themselves synthesized on the ribosome). RNA functions as both catalyst (*e.g.* in peptide synthesis and tRNA maturation) and genome (in RNA viruses such as HIV and influenza viruses). In contrast to nucleic acids, which associate according to the rules of base pair complementarity, the intricacies of protein structure do not – normally – allow for an easy mechanism of replication, which presumably explains the evolution of a coded system for their synthesis (for an interesting discussion of the contrasting molecular requirements for replication and catalysis, see [12]). Parsimony at least would seem to favour a scenario in which functions carried out by two classes of macromolecules in the modern system were, at an earlier stage, carried out by only one (for an alternative view however, see [13]). So which came first, the chicken or the egg? Protein or RNA? This is an underlying current in the debate surrounding the RNA world hypothesis, which I address when I discuss the ‘proteins first’ hypothesis.

Before beginning, it is important to clear up a common source of confusion. The RNA world hypothesis does not necessarily imply that RNA was the first replicating molecule to appear on the Earth (although a new paper by Benner and colleagues argues that this was, in fact, the case [14]). The more general claim is that the RNA world comprised a stage of evolution preceding – perhaps immediately – the RNA/protein/DNA world we now inhabit. In this way, the hypothesis is not incompatible with models such as the ‘crystals-as-genes’ concept of Cairns-Smith [15], which proposes that the first replicators were imperfection-containing layers of clay that were able to pass on these imperfections to proceeding layers (unfortunately, one experimental test of Cairns-Smith’s model suggests that replicated defects are quickly overrun by random defects or noise [16]). Similarly, it has been hypothesized that RNA was preceded in evolution by a nucleic acid analogue – for example, one in which glycerol replaces ribose in the phosphodiester backbone – though pathways for the prebiotic synthesis of many such analogues are even less plausible than for RNA itself [17].

## Discussion

The following objections to the RNA world hypothesis have been raised:

### RNA is too complex a molecule to have arisen prebiotically

RNA is an extremely complex molecule, with four different nitrogen-containing heterocycles hanging off a backbone of alternating phosphate and D-ribose groups joined by 3′,5′ linkages. Although there are a number of problems with its prebiotic synthesis, there are a few indications that these may not be insurmountable. Following on from the earlier work of Sanchez and Orgel [18], Powner, Sutherland and colleagues [19] have published a pathway for the synthesis of pyrimidine nucleotides utilizing plausibly prebiotic precursor molecules, albeit with the necessity of their timed delivery (this requirement for timed delivery has been criticized by Benner and colleagues [14], although most origin of life models invoke a succession of changing conditions, dealing as they do with the evolution of chemical systems over time; what is critical is the plausibility of the changes). A particularly interesting aspect of the pathway is the use of UV light as a method of isolating the naturally occurring nucleotides [18,19], suggesting a possible means of nucleotide selection (see also [20]).

Although RNA is constructed with uniform 3′,5-linked backbones, recent work by Szostak and colleagues has demonstrated that ribozymes and RNA aptamers retain partial function when the standard 3′,5′-linkages are replaced with a mixture of 3′,5′- and 2′,5′- linkages, suggesting that a degree of heterogeneity may be compatible with (or even beneficial to) RNA function and synthesis (J. Szostak, pers. commun.; [21]). This complements an earlier study by Ertem and Ferris [22] that showed that poly C oligonucleotides with mixed 3′,5′- and 2′,5′-linkages are able to serve as templates for the synthesis of poly G oligonucleotides by nonenzymatic replication. Such work suggests that ancestral systems may not have been as tightly constrained as they are today.

Due perhaps to the molecular complexity of nucleic acids, metabolism-first models (as opposed to replication-first models such as the RNA world hypothesis) highlight the importance of the initial generation of small molecules through chemical or metabolic cycles. Establishment of a plausible energy source is a critical aspect of these models, some of which propose that life arose in the vicinity of hot alkaline (pH 9–11) under-sea hydrothermal vents, with energy provided by pH and temperature gradients between the vent and the cooler, more acidic ocean [23-26]. In some ways, metabolism-first models appear not to conflict with the RNA world hypothesis, as they potentially offer a solution to the difficulty of ribonucleotide and RNA synthesis. A large point of difference, however, comes with the claim that such nucleic acid-free systems are capable of Darwinian evolution. Addressing this claim, Vasas *et al.*[27] have reported a lack of evolvability in such systems, while Benner and colleagues have noted the lack of experimental support from specific chemical models [14]. A more recent paper by Vasas *et al.*[28], while seemingly contradicting their earlier paper, uses a computational modeling approach without reference to a real-world chemical system (something noted by two of the reviewers in their published reviews).

### RNA is inherently unstable

RNA is often considered too unstable to have accumulated in the prebiotic environment. RNA is particularly labile at moderate to high temperatures, and thus a number of groups have proposed the RNA world may have evolved on ice, possibly in the eutectic phase (a liquid phase within the ice solid) [29-33]. Two of these studies [31,32] demonstrated maximal ribozymic activity at −7 to −8°C, possibly due to the combined effects of increased RNA concentration and lowered water activity. A possible difficulty with this scenario is that RNA sequences have an increased tendency to base pair at such temperatures, leading in some cases to the formation of intermolecular complexes [34] that potentially could reduce catalytic activity.

A further problem is the susceptibility of RNA to base-catalyzed hydrolysis at pH >6 [35]. The phosphodiester bonds of the RNA backbone and the ester bond between tRNAs and amino acids – something similar to which would have been critical for the evolution of ribosomal protein synthesis – are both more stable at pH 4–5 [5,6]. With our proposal for RNA world evolution at acidic pH [5], we have suggested that the primordial ‘soup’ may have been more like vinaigrette, while Hanczyc [36] has drawn a comparison with mayonnaise, with its emulsified mixture of oil in water (in light of these, could there be potential for food science to provide insights for origin of life studies?) While Mg^2+^ is important for stabilizing RNA secondary and tertiary structure, high Mg^2+^ concentrations also catalyze RNA degradation, which has been identified as a particular problem in the case of RNA template copying [21]. Here too, acidic pH offers a possible solution, as the positive charge on protonated cytosine and adenosine residues in acidic conditions may reduce the requirement for divalent cations. For example, a self-cleaving ribozyme with maximum activity at pH 4 isolated by *in vitro* selection, is active in the absence of divalent ions (including Mg^2+^) [37]. RNA secondary (and tertiary) structure would appear to be compatible with the presence of protonated nucleotides, as we have found an increased number of potentially protonated A-C base pair ‘mismatches’ in the tRNAs from acidophilic archaeal species with reported cytoplasmic pHs of 4.6-6.2 [5].

### Catalysis is a relatively rare property of long RNA sequences only

The RNA world hypothesis has been criticized because of the belief that long RNA sequences are needed for catalytic activity, and for the enormous numbers of randomized sequences required to isolate catalytic and binding functions using *in vitro* selection. For example, the best ribozyme replicase created so far – able to replicate an impressive 95-nucleotide stretch of RNA – is ~190 nucleotides in length [38], far too long a sequence to have arisen through any conceivable process of random assembly. And typically 10,000,000,000,000-1,000,000,000,000,000 randomized RNA molecules are required as a starting point for the isolation of ribozymic and/or binding activity in *in vitro* selection experiments, completely divorced from the probable prebiotic situation. As Charles Carter, in a published review of our recent paper in *Biology Direct*[5], puts it: 

“I, for one, have never subscribed to this view of the origin of life, and I am by no means alone. The RNA world hypothesis is driven almost entirely by the flow of data from very high technology combinatorial libraries, whose relationship to the prebiotic world is anything but worthy of “unanimous support”. There are several serious problems associated with it, and I view it as little more than a popular fantasy” (reviewer's report in [5]).

10^14^-10^16^*is* an awful lot of RNA molecules. However, the discovery of a number of extremely short ribozymes suggests that long sequences – and hence the huge numbers of RNA molecules required to sample the necessary sequence space – might not have been necessary. In a section titled ‘Miniribozymes: small is beautiful, Landweber and colleagues [31] discuss a number of such small ribozymes, including a minimal size active duplex of only 7 nucleotides that self-cleaves. Regarding the relatively modest rate enhancement of this miniribozyme – three orders of magnitude less than the parent ribozyme from which it is derived – the authors conclude: “the smallest molecules are likely to arise first, and any rate enhancement would have been beneficial in a prebiotic setting” [31]. Another, closely related, miniribozyme can ligate a small RNA to its 5′ end, requiring only a single(!) bulged nucleotide in the context of a larger base-paired structure containing a strand break. Interestingly, the self-cleaving 7-nucleotide sequence forms a part of the ligase ribozyme, demonstrating the closeness in sequence space of the two, albeit related, functions [31]. Equally as interesting from an RNA world perspective, Yarus and colleagues have recently isolated by *in vitro* selection a ribozyme that is able to be truncated to just 5 nucleotides, while retaining its ability to catalyze the aminoacylation *in trans* of a 4-nucleotide RNA substrate [39]. Remarkably, only 3 nucleotides are responsible for this activity: 2 in the ribozyme and 1 in the substrate. In fact, even this much is not required: a variant of the parent ribozyme with a mutation of 1 of the 3 conserved nucleotides is able to aminoacylate a substrate variant with the sequence GCCA (similar to the universal aminoacylated 3′ terminus of tRNA), albeit at a reduced rate [40] (we have previously proposed a possible sequence for an aminoacylating ribozyme based on this variant that could have base-paired with the universal 3′ CCA termini of tRNAs (and proposed RNA hairpin precursors [41,3] through a double helix interaction, while also forming *specific* triple helix interactions – at acidic pH – with other nucleotides in the tRNA [5]). As with the small ribozymes discussed by Landweber and colleagues, the rates of aminoacylation of Yarus' ribozymes are somewhat underwhelming: that of the original 5-nucleotide ribozyme is only 25-fold higher than the uncatalyzed rate [39], while that of the variant is only 6-fold higher than the uncatalyzed rate [40] (for further discussion of the implications of such tiny ribozymes see [42], and [31] and references therein).

**Figure 1:**
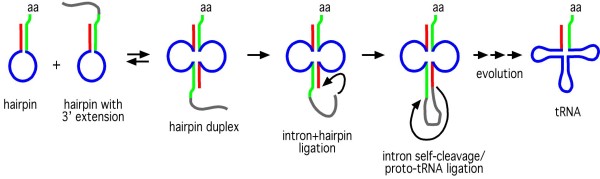
**A proposal for the origin of tRNA through the ligation of a hairpin duplex catalyzed by an ancestral self-splicing group I-type intron based on proposals by Di Giulio **[41]**, and Dick and Schamel **[49]**.** In this depiction, the intron is shown as originating from a 3′ extension of one of the precursor hairpins formed by a transcriptional runoff error. aa indicates the amino acid binding site, but is not meant to imply that an amino acid was necessarily attached here during the intron ligation events.

Although not quite as small as the ribozymes discussed above, Gross and colleagues have demonstrated that 12-nucleotide and 20-nucleotide nuclear tRNA^Tyr^ introns from *Arabidopsis thaliana* and *Homo sapiens* – understood to be cleaved by protein enzymes *in vivo* – are able to self-cleave in the presence of 10 mM Mg^2+^, 0.5 mM spermine and 0.4% Triton X-100 [43-45]. Although the introns form part of a larger pre-tRNA sequence, the nucleotides responsible for self-excision are possibly confined to a 3- or 4-nucleotide bulge region. The discovery of this intrinsic activity (which admittedly requires the presence of a low concentration of surfactant) supports previous proposals for the origin of tRNA [41,3,4]. Although there exist a number of other models for the origin of tRNA (one of which is discussed in detail in the following section), a hairpin duplication-ligation origin stands as a credible hypothesis [41,3] that has received support from a number of sources [46-48]. Briefly, the idea - first proposed by Di Giulio [41] - is that two (either identical or very similar) hairpins, approximately half the size of contemporary tRNA, formed a ligated duplex due to the symmetry of base-pairing interactions, possibly by an intron-mediated mechanism [49] (Figure 1). It has been proposed previously that contemporary protein-spliced nuclear tRNA introns are descended from an ancestral self-splicing group I-type intron that catalyzed the ancestral ligation [49] (as depicted in Figure 1, the ancestral tRNA intron may have derived from a 3′ extension of one of the precursor hairpins by a transcriptional runoff error). The findings of Gross and colleagues [43-45] indicate that some normally protein-cleaved nuclear tRNA introns have partially retained the ability to self-cleave. This ability to self-cleave implies the reverse reaction – self-ligation – is also possible, which could have produced the ligated intron-containing hairpin intermediate; subsequent intron self-cleavage could have produced the first proto-tRNA [49] (Figure 1). 

### The catalytic repertoire of RNA is too limited

It has been suggested that the probable metabolic requirements of an RNA world [50] would have exceeded the catalytic capacity of RNA. The majority of naturally occurring ribozymes catalyze phosphoryl transfer reactions – the making and breaking of RNA phosphodiester bonds [51]. Although the most efficient of these ribozymes catalyze the reaction at a comparable rate to protein enzymes – and *in vitro* selection has isolated ribozymes with a far wider range of catalytic abilities [9,51] – the estimate of proteins being one million times fitter than RNA as catalysts seems reasonable, presumably due to proteins being composed of 22 chemically rather different amino acids as opposed to the 4 very similar nucleotides of RNA [12].

It is frequently forgotten however that proteins too have their catalytic limitations: after all, many enzyme active sites contain cofactors and/or coordinated metal ions, suggesting that some reactions are ‘too hard’ for proteins as well (it is estimated that ~50% of proteins are metalloproteins [52], although of course not all these metal ions are found at the active site). RNA riboswitches bind a range of protein cofactors, such as flavin mononucleotide, thiamine pyrophosphate, tetrahydrofolate, *S-*adenosylmethionine and adenosylcobalamin (a form of vitamin B12) [53]. In the case of the *glmS* riboswitch/ribozyme, the metabolite glucosamine-6-phosphate binds in the active site and appears to participate in catalysis [54]. Because of the ability of these naturally occurring RNA riboswitches to bind protein enzyme cofactors, and because many of these cofactors possess non-functional fragments of RNA – one of the earliest pointers to a possible ancestral RNA world [55] – it is likely that at least some of the cofactors now used by proteins were handed down directly from the RNA world, where they played a similar if not identical role in assisting catalytic function [53].

One of the arguments for the RNA world hypothesis comes from the observation that RNAs are, in most cases, worse catalysts than proteins. This implies that their presence in modern biological systems can best be explained by their being remnants of an earlier stage of evolution, which were too embedded in biological systems to allow replacement easily. An alternative explanation is that they were co-opted by a protein world due to their superior properties for the particular functions they perform. While such an explanation seems intuitively less likely, surprisingly it is held by some proponents of the ‘proteins first’ model [56-60] (discussed in more detail below).

### Proteins first

An increasingly strident view is that protein either preceded RNA in evolution or, at the very least, that RNA and protein coevolved, in what is known as the ‘proteins (or peptides) first’ hypothesis [56-60]. Take, for example, Charles Kurland in his 2010 piece in *Bioessays*[57], which is utterly scathing of the RNA world hypothesis and its fellow travelers: 

“[The RNA world hypothesis] has been reduced by ritual abuse to something like a creationist mantra”, and

“[The] RNA world is an expression of the infatuation of molecular biologists with base pairing in nucleic acids played out in a one-dimensional space with no reference to time or energy” [57].

On a less emotional note, Harish and Caetano-Anollés [60] earlier this year published a phylogenetic analysis of ribosomal RNA and ribosomal proteins, concluding that the oldest region of the ribosome is a helical stem of the small ribosomal subunit RNA and the ribosomal protein that binds to it. As this helical stem has the important roles in the modern ribosome of decoding the mRNA message and in the movement of the two subunits relative to each other (including translocation of the mRNA message and tRNAs), Harish and Caetano-Anollés conclude that the original function of the ribosome was as an RNA replicase (this idea, which has been suggested previously, is discussed in detail in the following section). In addition, because RNA and protein components of the ribosome apparently have similar ages, Harish and Caetano-Anollés surmise that peptide synthesis has always been carried out by RNA in association with proteins, as is the case with the modern ribosome.

Without debating the merits or otherwise of their phylogenetic techniques, the most serious objection to these conclusions is that phylogenetic analysis has the limitation that it can only analyze the protein sequence record as it has been captured in DNA (this is true even for a phylogenetic analysis based on protein fold structures, as the only record we possess of these folds is their primary amino acid sequence as captured in the DNA). Therefore, any information we can recover can only date from the advent of coded protein synthesis, as that is the point at which protein sequence became coded in nucleic acid. In an online report [61] on Harish and Caetano-Anollés’ paper, Russell Doolittle makes this same point:

“This is a very engaging and provocative article by one of the most innovative and productive researchers in the field of protein evolution,” said University of California at San Diego research professor Russell Doolittle, who was not involved in the study. Doolittle remains puzzled, however, by “the notion that some early proteins were made before the evolution of the ribosome as a protein-manufacturing system.” He wondered how – if proteins were more ancient than the ribosomal machinery that today produces most of them –“the amino acid sequences of those early proteins were ‘remembered’ and incorporated into the new system.” [61].

To which, Caetano-Anollés’ reported response is slightly puzzling:

“It requires understanding the boundaries of emergent biological functions during the very early stages of protein evolution. However, the proteins that catalyze *non-ribosomal* protein synthesis – a complex and apparently universal assembly-line process of the cell that does not involve RNA molecules and can still retain high levels of specificity – are more ancient than ribosomal proteins. It is therefore likely that the ribosomes were not the first biological machines to synthesize proteins.” ( [61]; italics in original).

It is certainly possible that there were functional noncoded peptides prior to the advent of coded protein synthesis. These could have been formed either through random processes, by noncoded ribosomal synthesis prior to the advent of coding [4], by non-ribosomal peptide synthesis catalyzed by specific ribozymes (analogous to non-ribosomal peptide synthesis catalyzed by protein enzymes in modern systems [62]), or by some combination of the above. It seems highly unlikely, however, that proteins synthesized proteins prior to the advent of the ribosome, as this would appear to suggest an infinite regression series. As Doolittle [61] suggests, the critical point is that once coding evolved, the sequences of these noncoded proteins would have needed to be recapitulated by coded proteins; therefore the phylogenetic signal would only go back to the point of recapitulation. Put another way, the earliest proteins phylogenetically speaking will be the first proteins that were coded for. Presumably, if these sequences can still be detected in modern genomes, they would tend to be relatively short and somewhat indistinct traces only, as one might expect for the first proteins produced by a rudimentary ribosome. In a sense then, one can say that the advent of coded protein synthesis has drawn a veil over the previous life of proteins. Although it seems unlikely, complex proteins *may* have existed prior to this, but – as all record of them has been erased by the advent of coding – that is as much as we can say (for an in-depth discussion of the implications of non-ribosomal peptide synthesis for the RNA world hypothesis, see [62]).

### RNA replicase origin of the ribosome

As mentioned above, Harish and Caetano-Anollés are not the first to suggest an RNA replicase origin of the ribosome (or small ribosomal subunit). The idea, which was possibly first proposed by Weiss and Cherry [63], is that “the ancestor of small subunit RNA was an RNA replicase that used oligonucleotides as a substrate” [63]. The hypothesis has grown in scope to include the use of excised tRNA anticodons as the source of oligonucleotides, with the energy required for ligation provided by concomitant peptide bond formation [64-66]. However, as pointed out by Wolf and Koonin [67], such a ligase would have required a molecular machinery at least as complex as the modern ribosome, which would make it an unlikely evolutionary forerunner. This notwithstanding, Weiss and Cherry’s original, simpler, model may have some merit. If, as has been recently suggested, early RNA replication was performed by the ligation of short oligonucleotides [68,69], or by a combination of nucleotide polymerization and oligonucleotide ligation [21], a ‘decoding’ RNA able to proofread triplet base pair interactions for accuracy – similar to its role in the modern ribosome of maintaining the fidelity of the triplet codon-anticodon interaction – might have played an important role. Interestingly, a 49-nucleotide hairpin comprising part of the decoding site of the small ribosomal subunit RNA has been found to bind both poly U oligonucleotide and the tRNA^Phe^ anticodon stem-loop in a similar fashion to the entire small subunit [70]. This hairpin contains the two mobile nucleotides A_1492_ and A_1493_ (numbered according to the *Escherichia coli* small ribosomal subunit RNA sequence) that proofread the anticodon-codon helix in the modern ribosome [71]. It would be interesting to test whether this hairpin is able to enhance the rate and/or accuracy of non-enzymatic ligation using a single-stranded RNA ‘template’ and short complementary oligonucleotides. If an enhancement were indeed demonstrated, such a mechanism would be analogous to that utilized by the large ribosomal subunit, for which substrate positioning of the two tRNAs may constitute one of its main roles in catalyzing peptide synthesis [72].

As part of their model of early RNA replication by oligonucleotide ligation, Manrubia and colleagues propose that an increase in the catalytic rate of the replicase/ligase would have occurred with an increase in sequence length through a process of bootstrapping [68,69]. Furthermore, they suggest that the first RNA replication possibly had a high error-rate: 

“Highly mutagenic replication processes could have produced relatively large repertoires of short, genetically different molecules, some of them folding into secondary/tertiary structures able to perform selectable functions” [68].

Similarly, we have proposed that, in an RNA world evolving at acidic pH, non-standard base pairing interactions due to base protonation could have provided a means of increasing RNA sequence variation through non-enzymatic replication [5].

### The origin of tRNA

Wiener and Maizels’ genomic tag hypothesis proposes that the 3′ (or ‘top’) half of tRNA originally functioned as a tag demarking the 3′-end of genomic RNAs for replication, and thus was the first part of tRNA to evolve [73]. Sun and Caetano-Anollés [74,75] have published phylogenetic evidence that they believe supports the genomic tag hypothesis by confirming, “that the ‘top half’ of tRNA is more ancient than the ‘bottom half’” [75]. Noller [76] has observed that the tRNA top half (comprising the T arm and the acceptor stem – including the amino acid binding site) interacts almost exclusively with the large ribosomal subunit, while the bottom half (comprising the D and anticodon arms) interacts almost exclusively with the small subunit. Because peptide synthesis (a function of the large subunit) is usually viewed as more ancestral than decoding (a function of the small subunit) – a view which has support from a structural analysis by Bokov and Steinberg [77] – the top half of tRNA (which interacts with the large subunit) has been viewed as being more ancestral than the bottom half [73,78]. However, this ‘standard model’ for the origin of tRNA, and the results of Sun and Caetano-Anollés that support this model [74,75], are apparently both in conflict with Harish and Caetano-Anollés’ [60] more recent findings on the relative ages of the ribosomal subunits. As described above, these findings suggest that the small ribosomal subunit was the first to evolve, which is difficult to reconcile with the fact that the bottom half of tRNA (with which the small subunit mainly interacts), is, by theirs [74,75] and others [73,78] estimation, the newer half of tRNA. Equally, their finding that the large ribosomal subunit evolved more recently [60] is difficult to reconcile with the fact that the top half of tRNA (with which the large subunit mainly interacts), is, by theirs and others estimation, the older half of tRNA. Incidentally, Caetano-Anollés and colleagues’ finding [75,79,80] that the most ancient tRNAs coded for selenocysteine, tyrosine, serine and leucine not only runs counter to other work in the area (see *e.g.*[81]), but – as these tRNAs all possess long variable arms – appears to contradict their own finding that the “variable region was the last structural addition to the molecular repertoire of evolving tRNA substructures” [74].

 As discussed above, a plausible scenario for the origin of tRNA is the duplication and subsequent ligation of an RNA hairpin approximately half the length of modern tRNA (or alternatively the ligation of two very similar hairpins) [41,3], with ligation possibly catalyzed by an ancestral self-cleaving intron [49] (see Figure 1). An important implication of such an origin is that both tRNA halves are of equal antiquity, as both would have to be present for ligation to occur! However, due to the symmetry of the tRNA molecule, the top half, which is considered to be the more ancient, *is* in fact more ancient-*like*, as it retains the base-paired 3′ and 5′ ends of the original hairpin from which it derives. In contrast, the bottom half, considered to be the more recently acquired, contains the ‘join’ between the two hairpins, which has altered the conformation of the original hairpin, giving this bottom half a new structure. If one accepts a hairpin duplication-ligation origin of tRNA, this explains why the top half of tRNA interacts with the peptidyl transferase region of the large ribosomal subunit: it is because this half retains the same structure (and possibly nucleotide sequence) as the hairpin from which it derives, which originally interacted with the peptidyl transferase region of the large subunit. In fact - and this point has been made by others [49] – this retention of structure probably favoured (or even enabled) the duplication event, as it meant the resultant tRNA was able to be aminoacylated by the same ribozyme synthetase that aminoacylated the hairpin precursor, and therefore the tRNA was able to participate in ribosomal protein synthesis. At the same time, the appearance of a novel structure at the ligation point – the anticodon loop – allowed for the subsequent evolution of genetic coding [4,3].

One of the strongest arguments in favour of the hairpin ligation being catalyzed by an ancestral self-cleaving intron [49] (as depicted in Figure 1) is the presence of the highly conserved ‘canonical intron insertion position’ between nucleotides 37 and 38 in the anticodon loop [41], where almost all eukaryotic nuclear (and the majority of archaeal) tRNA introns are found, even though introns are only found in a subset of tRNA isoacceptors [82]. It has been proposed previously that this conserved position constitutes a 'molecular memory’ of the position of the ancestral intron that was responsible for the ligation that created the first tRNA [83]. If the canonical intron insertion position *is* ancestral, it implies that eukaryotic nuclear tRNAs (and possibly archaeal tRNAs) have a more ancestral structure than eubacterial tRNAs, which usually lack tRNA introns altogether or possess self-splicing introns at a variety of different positions in the molecule. Such a finding is consistent with the introns-early hypothesis, and the proposal that eubacteria have undergone a process of intron loss [84,85].

## Conclusions

I have argued that the RNA world hypothesis, while certainly imperfect, is the best model we currently have for the early evolution of life. While the hypothesis does not exclude a number of possibilities for what – if anything – preceded RNA, unfortunately the evolution of coded protein synthesis has drawn a veil over the previous history of proteins. The situation is different in the case of non-coding RNAs such as ribosomal RNA and tRNA, as these were able to replicate prior to the evolution of ribosomal protein synthesis.

As we have noted previously [5], the proposal that the RNA world evolved in acidic conditions [5,6] offers a plausible solution to Charles Kurland's criticism [57] that the RNA world hypothesis makes no reference to a possible energy source. As de Duve [87] has noted, "the widespread use of proton-motive force for energy transduction throughout the living world today is explained as a legacy of a highly acidic prebiotic environment and may be viewed as a clue to the existence of such an environment" [87]. Although Russell, Martin and others [23-26] have argued that proton and thermal gradients between the outflow from hot alkaline (pH 9-11) under-sea hydrothermal vents and the surrounding cooler more acidic ocean may have constituted the first sources of energy at the origin of life, the lack of RNA stability at alkaline pH ( [5] and references within) would appear to make such vents an unlikely location for RNA world evolution.

Although possible, it seems unlikely that the A-C base pair 'mismatches' found in the tRNA genes of Ferroplasma acidarmanus and Picrophilus torridus (two species of archaebacteria with a reportedly acidic internal pH) [5] are corrected by C to U RNA editing that occurs, for example, with some - but not other - plant chloroplast tRNAs [88,89]. Such editing of secondary structure A-C base pair mismatches has so far not been found to occur in archaebacteria; however, in a single archaeal species (Methanopyrus kandleri) a tertiary structure A-C base pair found in 30 of its 34 tRNAs undergoes C to U editing catalyzed by a cytidine deaminase CDAT8 [90]. M. kandleri is a unique organism that contains many 'orphan' proteins. CDAT8, which contains a cytidine deaminase domain and putative RNA-binding domain, has no homologues in other arachaeal species, including F. acidarmanus and P. torridus (L Randau, pers. commun.; [90]). Definitive proof, however, that the A-C base pairs in these two species are not modified would of course require e.g. cDNA sequencing of the tRNAs.

## Abbreviations

mRNA: messenger RNA; tRNA: transfer RNA.

## Competing interests

The author declares that he has no competing interests.

## Reviewers’ comments

Referee 1: Eugene Koonin

I basically agree with Bernhardt. The RNA World scenario is bad as a scientific hypothesis: it is hardly falsifiable and is extremely difficult to verify due to a great number of holes in the most important parts. To wit, no one has achieved bona fide self-replication of RNA which is the cornerstone of the RNA World. Nevertheless, there is a lot going for the RNA World (Bernhardt summarizes much of the evidence, and I add more below) whereas the other hypotheses on the origin of life are outright helpless. Moreover, as argued in some detail elsewhere [91], the RNA World appears to be an outright logical inevitability. ‘Something’ had to start efficiently replicating to kick off evolution, and proteins do not have this ability. As Bernhardt rightly points out, it is not certain that RNA was the first replicator but it does seem certain that it was the first ‘good’ replicator. To clarify, this does not imply that the primordial RNA World did not have peptides; on the contrary, it is plausible that peptides played important roles but they were not initially encoded in RNA.

Moreover, straightforward observations on modern proteins indicate that the role of RNA in the ancient translation system was much greater that it is in the modern system. Indeed, Class I aminoacyl-tRNA synthetases (aaRS) represent only a small branch on the complex evolutionary tree of Rossmann-like domains, so the common ancestor of all 10 Class I aaRS emerged after extensive diversification of this particular class of protein domains had already taken place. Accordingly, one is compelled to conclude that a high-fidelity translation system that alone would enable extensive protein evolution existed already at the late stages of the hypothetical RNA World [92].

All this discussion is not pointless play with hypotheses. Realization of the unique status of the RNA World among the origin of life scenarios is critical for maintaining the focus of research on truly important directions such as experimental and theoretical study of the evolution of ribozymes rather than futile attempts to debunk the RNA World.

Referee 2: Anthony Poole

Harold Bernhardt’s review of the RNA world hypothesis is readable and timely. He presents a very open-minded review of recent results and how they impact on old ideas, and distills a large amount of material. Aside from the admirable attempt to synthesize a vast array of ideas, a valuable contribution hidden within is the critical assessment of the view that the RNA world hypothesis needs to be abandoned in favour of a peptides-first model.

Author’s response: *I have revised the abstract and introduction to include reference to my critique of the ‘proteins (or peptides) first’ hypothesis.*

*While I doubt that anyone seriously excluded peptides as part of a prebiotic milieu, the primacy of peptides does need careful consideration. In this regard, the explicit explanation of why a pre-genetic code origin of proteins will not be detectable from comparative genomic analyses is an important contribution. Perhaps this is obvious to some, but in light of a growing view that non-ribosomal peptide synthesis preceded ribosomal peptide synthesis, it would seem that the community needs a reminder, and Bernhardt spells it out in a very informative manner. Another issue with arguing for non-ribosomal peptide synthesis preceding the ribosome is that there is an enormous difference in information input versus output. As discussed in[*[62]*], megaenzymes like cyclosporin are ~15000 amino acids in length and produce products of 11 amino acids in length – a factor of 10*^*4*^*is not trivial. While non-ribosomal peptide synthetases are modular and could in principle be engineered into minimal entities, the challenge of equalizing information input and output is significant regardless of one’s favoured prebiotic starting point. It is clear from reading Bernhardt’s review that the RNA community is much closer to this than those who seek to replace primordial RNA-based replication with peptide-based replication.*

Referee 3: Michael Yarus (nominated by Laura Landweber)

Almost always, progress to new understanding is sporadic, with insights coming in separated locales. Difficulties temporarily immobilize discussion, but then are surmounted by a successful theory. This sometimes inchoate stagger toward a broader, more self-consistent argument is all that can be expected, even of an ultimately successful idea. Discussions of the RNA world sometimes forget this, and demand e.g., the ultimate replicase today! But this essay by Harold Bernhardt remembers what has happened for other successful evolutionary ideas, like the big tree. For all its successes, the tree is still being questioned under extreme prejudice in certain quarters, as is the RNA world.

Contrariwise, here we have here a sympathetic review of the support for the RNA world, which specifically makes the point that it fits our descent better than other ideas (You look like the son of a montmorillonite to me, ya mangy mutant!). It will be useful to those who want an entry to the RNA world literature, and could easily serve as the crux of a university course.

However, this is also its weakness; the text is polite and respectful, even to those whose ‘contribution’ has been otherwise. It treats even loony ideas (‘we need proteins to evolve translation!’) with deference. Or to put it in other words, it is edgeless – some attitude would be welcome. Some choice between hypotheses should go with the territory; some consequent make-or-break predictions are the responsibilities of a guide. But as a gentle introduction, you will not find better.

Author’s response: *In revising the manuscript, I have – to some degree inadvertently – added a bit more bite!*
